# Isolation and Properties of the Bacterial Strain *Janthinobacterium* sp. SLB01

**DOI:** 10.3390/microorganisms10051071

**Published:** 2022-05-23

**Authors:** Lubov Chernogor, Kseniya Bakhvalova, Alina Belikova, Sergei Belikov

**Affiliations:** Limnological Institute, Siberian Branch, Russian Academy of Sciences, 664033 Irkutsk, Russia; xenia_titova@mail.ru (K.B.); kolobok136@rambler.ru (A.B.)

**Keywords:** *Lubomirskia baicalensis*, *Janthinobacterium* sp. SLB01 strain, violacein, pigment, biofilm

## Abstract

Bacteria of the genus *Janthinobacterium* are widespread in soils and freshwater ecosystems and belong to the phylum Proteobacteria. The *Janthinobacterium* sp. SLB01 strain was isolated from diseased freshwater *Lubomirskia baicalensis* (Pallas, 1776) sponge, and the draft genome was published previously. However, the properties of the SLB01 strain are not known. The aim of the study is to describe some properties of the *Janthinobacterium* sp. SLB01 strain, isolated from *L. baicalensis* sponge. The identification of the SLB01 strain was established as Gram-negative, motile, rod-shaped, and psychrotolerant, with growth at 3 and 22 °C. We found that the SLB01 strain has proteolytic, lipolytic, and saccharolytic activity and can use citrates and reduce nitrates. The bacteria *Janthinobacterium* sp. SLB01 strain can grow, form biofilms, and produce the violet pigment violacein. We identified the pigments violacein and deoxyviolacein by chromatography and mass spectrometry. These metabolites may be of interest to biotechnology in the future. The studied characteristics of the *Janthinobacterium* sp. SLB01 strain are an important addition to previous studies of the genome of this strain. This study will help us to understand the relationship between the microbial communities of Lake Baikal and sponges.

## 1. Introduction

The bacteria of the genus *Janthinobacterium* are widespread and belong to the phylum *Proteobacteria* of the family *Oxalobacteraceae* [1,2,3]. Many authors have described the *Janthinobacterium* isolated from different environments, most commonly from soil and water ecosystems in cold temperatures, including freshwater ecosystems [4,5,6,7]. The *Janthinobacterium* are a Gram-negative, psychrotolerant, rod-shaped bacteria, motile by means of flagella [3,8]. It is known that *Janthinobacterium* can produce the purple pigment violacein, which has antibacterial, antiviral, antifungal, antitumor, antiparasitic, and antioxidant activity [9,10,11,12,13,14,15,16,17,18,19,20,21,22,23,24,25].

The *Janthinobacterium* can form biofilms on abiotic and biotic surfaces in aquatic ecosystems and can colonize fungi and fish skin [11,26,27,28,29,30]. Many researchers have shown that different bacterial species of the genus *Janthinobacterium* can be pathogens, e.g., *Janthinobacterium lividum* for the rainbow trout and *Janthinobacterium agaricidamnosum*, which can cause soft rot disease in the *Agaricus bisporus* mushroom [27,29,31].

For the first time, we isolated the *Janthinobacterium* sp. SLB01 strain from the diseased freshwater *L. baicalensis* sponges and sequenced its complete genome [32]. Since 2011, there has been a problem of the disease and death of freshwater endemic sponges in Lake Baikal [33,34]. Previously, we reported an increased abundance of microorganisms of the phyla *Proteobacteria* and the family *Oxalobacteraceae* in diseased *L. baicalensis* sponges and the infected cell cultures of primmorphs [34,35]. We carried out a genomic analysis comparing the *Janthinobacterium* sp. SLB01 strain to genomes of other related strains and revealed that it was closely related to the *J. lividum* MTR strain [20,36]. The aim of the study is to describe some properties of the *Janthinobacterium* sp. SLB01 strain, isolated from diseased *L. baicalensis* sponge. This study will expand our understanding of the relationship between the bacteria *Janthinobacterium*, with their properties, and the endemic freshwater sponges of Lake Baikal.

## 2. Materials and Methods

### 2.1. Isolation of the Janthinobacterium sp. SLB01 Strain

The *Janthinobacterium* sp. SLB01 strain was isolated earlier from a sample of diseased *L. baicalensis* sponge collected from Lake Baikal, Central Siberia, Russia. The strain was preserved as a 20% (*v*/*v*) glycerol suspension at −70 °C. We cultivated the *Janthinobacterium* sp. SLB01 strain on three different nutrient media for determining bacterial growth. The nutrient media had the following composition: plate count agar (PCA) with 0.5% tryptone, 0.25% yeast extract, 0.1% glucose, 1.5% agar, and final pH 7.2 (HiMedia, Mumbai, India); R 2A agar (R 2A) with 0.05% yeast extract, 0.05% protease peptone, 0.05% tryptone, 0.05% glucose, 0.05% soluble starch, 0.03% sodium pyruvate, 0.03% K_2_HPO_4_, 0.005% MgSO_4_, 1.5 agar, and final pH 7.2 (HiMedia, Mumbai, India), and medium Luria–Bertani (LB) with 1.5% agar, 1% tryptone, 0.05% yeast extract, 0.05% NaCl, and final pH 7.2 (HiMedia, Mumbai, India). The dishes were inoculated in three repetitions and cultivated at a temperature of 22 °C for 5 days, observing the growth of strains daily.

Additionally, the bacteria were cultivated in LB broth at different temperatures of 3, 22, and 30 °C for 10 days, measuring growth daily (OD_600_) with a spectrophotometer (GBC Scientific Equipment Ltd.—Cintra 20, Melbourne, Australia). Cell morphology was determined by light microscopy (Olympus IX73SC, Olympus Corp., Tokyo, Japan). Gram staining was carried out using the Gram staining kit HiAssortedTM K001-1KT (HiMedia, Mumbai, India).

### 2.2. Pigment Extraction

The 300 µL liquid culture medium containing a bacterial biomass of the *Janthinobacterium* sp. SLB01 strain with a pigment was transferred to a 1.5 mL centrifuge tube; 200 µL n-butanol was added, mixed thoroughly, and kept for 15 min in an ultrasonic bath (Sonorex super, Bandelin GmbH & Co., Berlin, Germany). Each sample was centrifuged with the Eppendorf Microcentrifuge 5415R (Merck, Darmstadt, Germany) at 7000 rpm for 20 min. The violacein containing an n-butanol layer was transferred to another tube. The bottom portion containing cell debris and traces of pigment was separated and re-extracted again with 100 µL n-butanol to recover the trace of pigment. The n-butanol layers were pooled together and concentrated through drying in a vacuum concentrator (Concentrator plus, Eppendorf, Hamburg, Germany) at 30 °C. The concentrated n-butanol extract containing violacein and other soluble impurities was extracted in 400 µL chloroform and was passed through a 0.45 μm pore-sized filter. The pigment precipitate was removed from the filter in two portions of 100 μL of methanol for LC analysis and MS spectrometry.

Chromatographic separations were obtained using a microcolumn chromatographic system (Milichrom-A02, Ltd Institute of Chromatography «EcoNova», Novosibirsk, Russia) with a Nucleosil 5-C18 column (75 × 2 mm, 1.7 μm). Gradient elution was carried out using the following solvent systems: phase A (Milli-Q water + 0.1% formic acid) and phase B (acetonitrile + 0.1% formic acid). The solvent gradient programming was 5% B in 8 min, and then from 5% to 100% B at a flow rate of 100 μL min^−1^, returning to the initial conditions in 2 min. The run time was 23 min per sample. The violacein methanol solution was characterized using UV–vis and electron spray ionization coupled with mass spectrometry (Ultraflex, Bruker Daltonik GmbH, Bremen, Germany).

### 2.3. Violacein Production

The *Janthinobacterium* sp. SLB01 strain was grown in 12-well plates (TPP Techno Plastic Products AG, Trasadingen, Switzerland) with LB broth at temperatures of 3, 22, and 30 °C to assess the accumulation of violacein. Violacein was extracted according to the previously described technique [11]. Bacterial cells from a single well were harvested by centrifugation at 16,000× *g* for 15 min every 24 h. The cells were lysed with 10% sodium dodecyl sulfate (*v*/*v*) (AppliChem, Darmstadt, Germany), and incubated at room temperature for 5 min. Then, water-saturated butanol (1:2) was added and shaken. The upper phase, containing violacein, was separated from the aqueous phase by centrifugation at 16,000× *g* for 10 min. The extracted violacein was quantified using a spectrophotometer (OD_585_) (GBC Scientific Equipment Ltd.—Cintra 20, Melbourne, Australia).

### 2.4. Biofilm Detection

We estimated the biofilm formation of the *Janthinobacterium* sp. SLB01 strain grown in 12-well plates (TPP Techno Plastic Products AG, Trasadingen, Switzerland) in LB broth at temperatures of 3, 22, and 30 °C. The biofilm amount was determined at regular time intervals according to a previously described method [11]. The wells of the plate were carefully emptied and washed twice with sterile 0.01 M phosphate-buffered saline (PBS) (Sigma-Aldrich, St. Louis, MO, USA) every 24 h. Then, 5 mL of an aqueous solution of 0.1% safranin (Sigma-Aldrich, St. Louis, MO, USA) was added to each well and incubated at room temperature for 20 min. The dye was removed with 0.01 M PBS buffer. Then, the plates were inverted onto filter paper (Macherey-Nagel, Düren, Germany) and dried for 3 h. Safranin was extracted with a mixture of ethanol 96% and acetone (80:20 *v*/*v*). The biofilm formation was quantified by measuring the optical density (OD_484_) of each sample using a spectrophotometer (GBC Scientific Equipment Ltd.—Cintra 20, Melbourne, Australia).

### 2.5. Biochemical Characterization of the Strain

The *Janthinobacterium* sp. SLB01 strain was analyzed using Hi Assorted Biochemical test kits for Gram-negative bacteria (HiMedia, Mumbai, India). We conducted biochemical tests for the determination of citrate utilization, L- lysine, L-ornithine, L-arginine, urease, phenylalanine deamination, nitrate reduction, hydrogen sulfide (H_2_S) production, glucose, lactose, arabinose, mannose, sucrose, adonitol, D-sorbitol, D-mannitol, and inositol. In addition, the SLB01 strain was tested using spirit blue agar, gelatin agar, and motility test medium (HiMedia, Mumbai, India). The test *Janthinobacterium* sp. SLB01 strain was grown in LB broth at 22 °C for 48 h and resuspended in the appropriate test media according to the manufacturer’s instructions, and 50 μL of the prepared inoculums was added to each well. The readings were performed after 48 h of incubation at 22 °C. The results were interpreted as per the standards given in the result chart.

### 2.6. Antibiotics Sensitivity Tests of the Strain

The *Janthinobacterium* sp. SLB01 strain was cultured on Mueller–Hinton agar (HiMedia, Mumbai, India) at 22 °C for 48 h and was tested with antimicrobial sensitivity discs of 20 antibiotics (HiMedia, Mumbai, India). The medium plates were prepared as per the manufacturer’s instructions. The following antibiotics were used in the study: ampicillin, ceftriaxone, imipenem, nalidixic acid, levofloxacin, lomefloxacin, norfloxacin, ofloxacin, ciprofloxacin, amikacin, gentalyn, gentamicin, kanamycin, netilmycin, tobramycin, doxycycline, tetracycline, chloramphenicol, erythromycin, and rifampicin. The results were analyzed as described in the manufacturer’s instructions.

### 2.7. Statistical Analysis

All the experiments were performed at least three times. The data were reported as means ± standard deviation (SD). The statistical analysis was carried out (single-factor (ANOVA) followed by Tukey’s multiple range test) using SPSS.16 software. Differences in mean values were considered significant at *p* < 0.05. All charts were built using Microsoft Office Professional Plus 2016 (Microsoft Corporation, Redmond, WA, USA).

## 3. Results

### 3.1. Isolation of the Bacterial Strains and Culture Conditions

**Figure 1:**
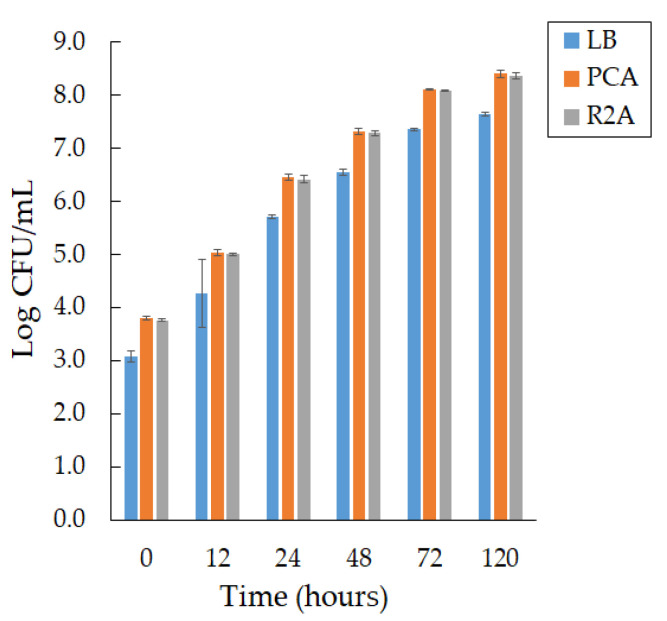
The growth of the *Janthinobacterium* sp. SLB01 strain at 22 °C on the different media (LB agar, PCA, and R 2A). Results are reported as mean values of three independent experiments performed.

We observed active growth during the formation of smooth, convex colonies with a purple pigment on days 2 and 3 of cultivation at a temperature of 22 °C on different media, PCA and R 2A, and a slight difference in bacterial growth in LB agar (Figure 1).

We found that the growth rate of bacteria of the *Janthinobacterium* sp. SLB01 strain does not depend on the composition of the medium; however, PCA medium is preferred.

**Figure 2:**
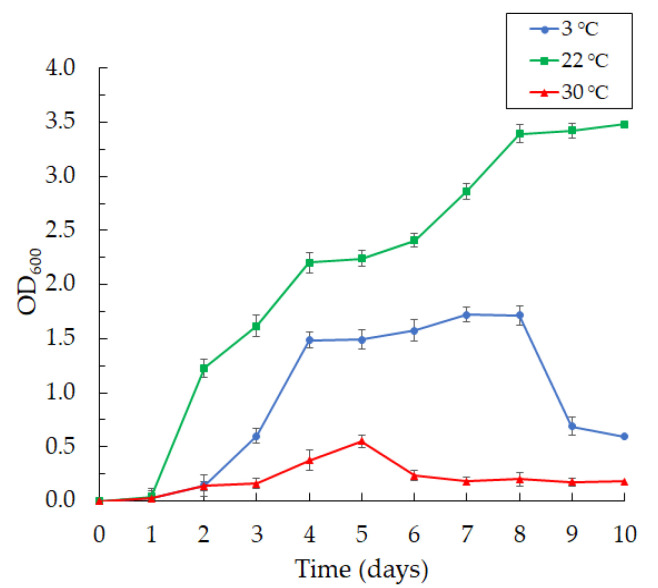
The curves of the *Janthinobacterium* sp. SLB01 strain at different temperatures of cultivation in LB broth (at 3, 22 and 30 °C). Results are shown as mean values of three independent experiments performed.

The bacteria were aerobic and psychrotolerant; they grew at 3 and 22 °C and did not grow well at 30 °C (Figure 2).

We found that the bacteria grew intensively on day 2 at 3 °C, reaching maximum growth by day 8 of cultivation, with a subsequent fall in growth.

**Figure 3:**
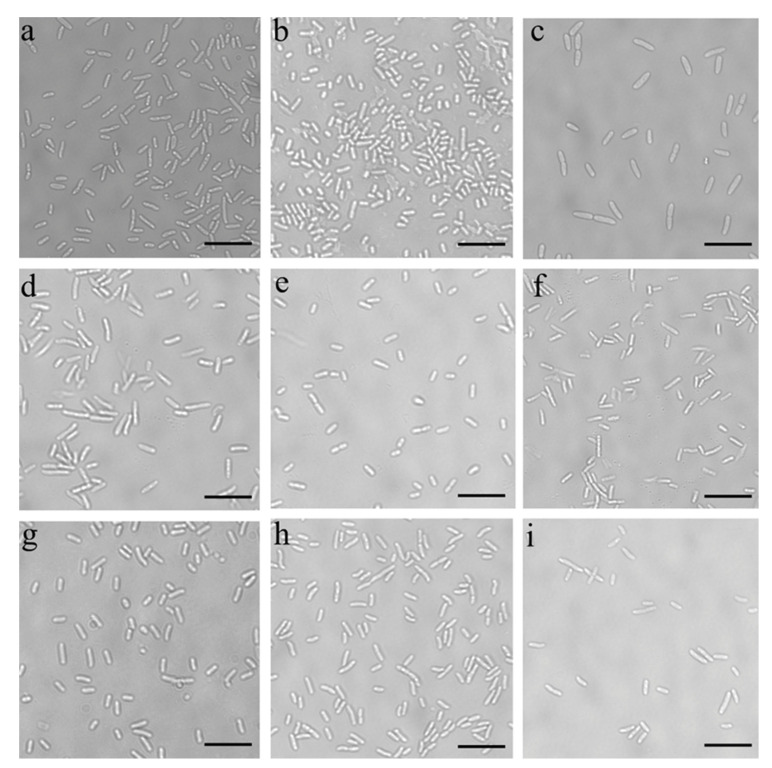
Light microscopy of the growth of the *Janthinobacterium* sp. SLB01 strain at different temperatures and in different media: (**a**) cultivation at 3 °C in PCA; (**b**) cultivation at 3 °C in R 2A; (**c**) cultivation at 3 °C in LB agar; (**d**) cultivation at 22 °C in PCA; (**e**) cultivation at 22 °C in R 2A; (**f**) cultivation at 22 °C in LB agar; (**g**) cultivation at 30 °C in PCA; (**h**) cultivation at 30 °C in R 2A; (**i**) cultivation at 30 °C in LB agar. Scale bar 10 µm.

The bacteria grew rapidly and produced violacein at a temperature of 22 °C. The bacteria of the *Janthinobacterium* sp. SLB01 strain were Gram-negative and did not form spores. They were rod-shaped with rounded ends and had a length of 1.0 to 5.0 µm and a width of 0.3 to 1.0 µm, with twitching motility. Moreover, we observed a change in the morphology of the bacteria during cultivation at different temperatures and media (Figure 3).

The bacterial cultures grew well at 22 °C in all the used nutrient media. However, we observed the formation of long cells in the *Janthinobacterium* sp. SLB01 strain at 30 °C.

### 3.2. Characterization of Violacein

**Figure 4:**
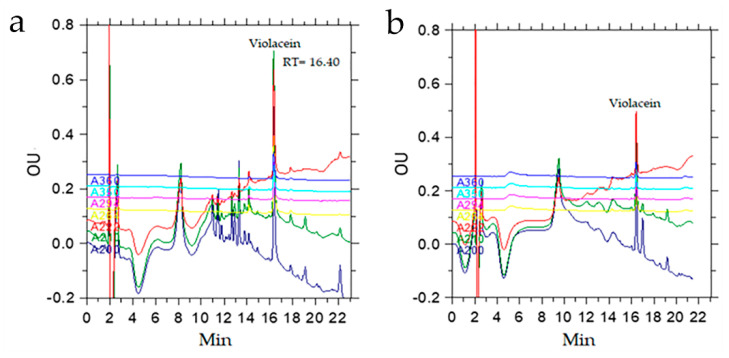
Characterization of the violacein of the *Janthinobacterium* sp. SLB01 strain extracted by different methods: (**a**) chromatogram of violacein using extraction with n-butanol; (**b**) chromatogram of violacein using extraction with chloroform. OU—optical density in optical units.

An extraction of 500 µL of the culture medium containing the biomass of the SLB01 strain with 300 µL of n-butanol allowed us to isolate more than 95% of the pigment. With repeated extraction, an additional 4% of the pigment can be isolated, as calculated from the ratio of the chromatographic peak areas. HPLC analysis data indicated that the butanol extract contained a peak with a retention time of 16.40 min, which was the bulk of the extract (53%), and the extract contained other substances with a lower retention time (Figure 4).

The presence of violacein and deoxyviolacein in the crude extract was confirmed by MALDY TOF MS analysis. The extract contained peaks of pseudomolecular ions with *m*/*z* 344 [M + H]+ and 328 [M + H]+, which belonged to violacein and deoxyviolacein, respectively. In addition, there was an ion with *m*/*z* 360 [M + H]+, corresponding to oxyviolacein [37].

### 3.3. Production of Violacein

**Figure 5:**
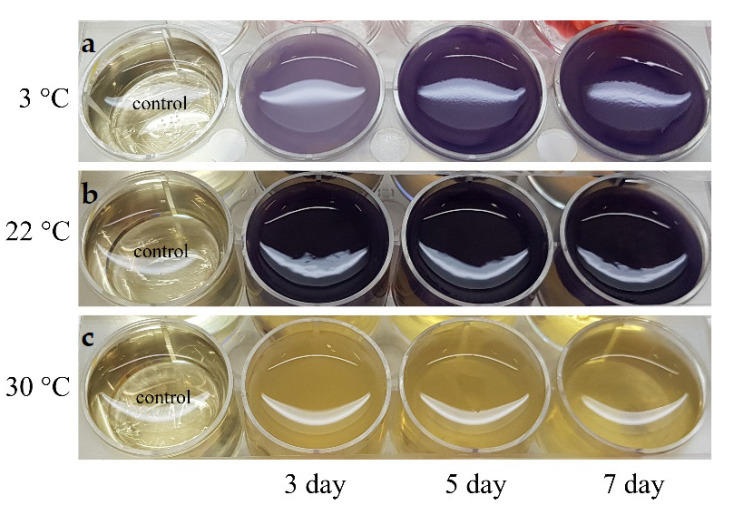
Production of violacein of the *Janthinobacterium* sp. SLB01 strain at different temperatures for five days. (**a**) Cultivation at 3 °C; (**b**) cultivation at 22 °C; (**c**) cultivation at 30 °C. The LB broth was taken as a control.

The cultivation of the *Janthinobacterium* sp. SLB01 strain in LB broth revealed the effect of different temperatures on the production of violacein (Figure 5).

**Figure 6:**
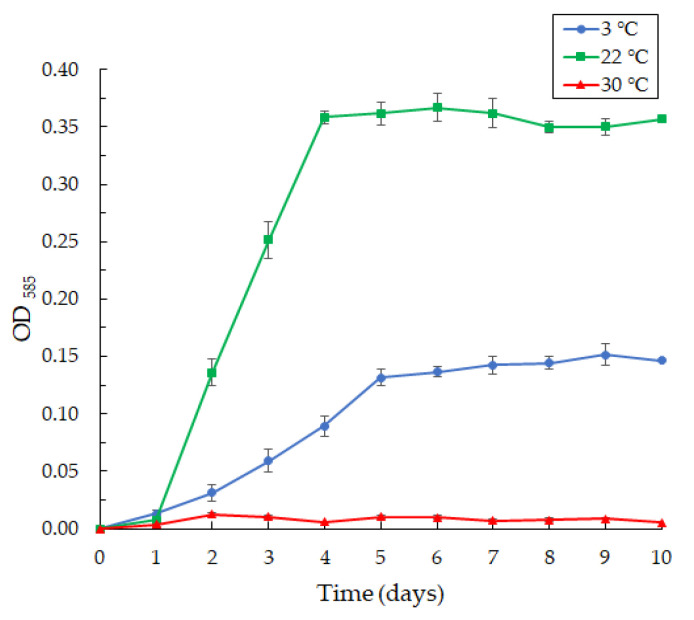
The curves of violacein production by the *Janthinobacterium* sp. SLB01 strain at 3, 22, and 30 °C in LB broth, OD_585_. Results are shown as mean values of three independent experiments performed.

The production of violacein was noted on the second day of cultivation, with intensive formation on the fifth day (Figure 6).

The *Janthinobacterium* sp. SLB01 strain was isolated from a diseased Baikal sponge collected earlier at a depth of 9–10 m and water temperature of 3–4 °C [32]. The SLB01 strain grew and produced violacein at 3 and 22 °C (Figure 6). However, the growth of the strain and violacein production was suppressed at 30 °C. We found that the SLB01 strain grew rapidly during the first five to ten days, with the simultaneous formation of violacein at 22 °C, reaching a maximum density (OD585) of 3.682; at a temperature of 3 °C, the concentration of violacein (OD585) reached 1.517.

### 3.4. Biofilm Formation

**Figure 7:**
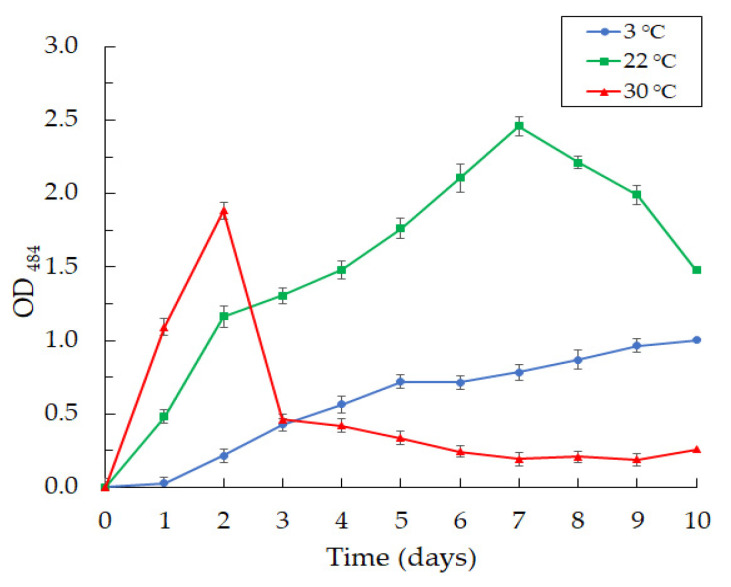
The curves of biofilm formation by *Janthinobacterium* sp. SLB01 strain at 3, 22, and 30 °C. Results are shown as mean values of three independent experiments performed.

Along with violacein production, we observed an intensive formation of biofilms at the cultivation of the *Janthinobacterium* sp. SLB01 strain at 3, 22, and 30 °C in LB broth medium (Figure 7).

We observed the intensive formation of dense biofilms in the first 24 h of cultivation at 22 and 30 °C. The highest OD_484_ index reached 1.303 at 22 °C and 1.937 at 30 °C for two days of cultivation. Then, the achieved OD_484_ index decreased sharply to 0.334 on the fifth day and to 0.256 on the tenth day of SLB01 cultivation at the temperature of 30 °C. The structure of the biofilms became loose and poorly attached to the walls of the plates. Interestingly, the indicators grew, and the OD_484_ index reached 2.560 at 22 °C for seven days and remained stable; the biofilms were dense. A completely different picture was observed at 3 °C. Biofilm formation gradually increased and reached the highest OD484 of 1.051 at 3 °C after 10 days of cultivation.

### 3.5. Biochemical Analysis

The biochemical characteristics of the *Janthinobacterium* sp. SLB01 strain are shown in Table 1.

Positive tests for the *Janthinobacterium* sp. SLB01 strain were as follows: citrate utilization, L-lysine, L-ornithine, L-arginine, nitrate reduction, lipolytic activity, glucose, sucrose, mannose, lactose, inositol, casein, and gelatin. Additionally, the test motility showed a positive reaction. Tests negative for the *Janthinobacterium* sp. SLB01 strain were urease production, phenylalanine deamination, H_2_S production, adonitol, arabinose, D-sorbitol, and D-mannitol.

### 3.6. Antibiotic Sensitivity Tests of the Strain

The antibiotic susceptibility test for studying the *Janthinobacterium* sp. SLB01 strain showed high resistance to multiple tested antibiotics (Table 2).

The SLB01 strain was resistant to ampicillin, imipenem, nalidixic acid, levofloxacin, norfloxacin, gentalyn, netilmycin, chloramphenicol, and erythromycin but susceptible to gentamicin, kanamycin, tobramycin, doxycycline, tetracycline, and rifampicin.

## 4. Discussion

Lake Baikal, located in the rift zone in the center of the Asian continent, is a cold-water lake [38]. The temperature of the water in the habitats of the Baikal sponges is within 3–4 °C. The mass diseases and death of freshwater sponges in Lake Baikal were observed in 2011 [33]. Subsequently, the disease of the Baikal sponges spread to the entire littoral of the lake. The study of the microbiomes confirmed the shift in the compositions of symbiotic bacteria in freshwater sponges [34,35]. Earlier, we showed that bacteria of the families *Oxalobacteraceae* and *Flavobacteraceae* increased in relative abundance in experiments on the infection of the cell culture of primmorphs [35].

The diseases and mass death of sea sponges are also observed in the seas and oceans and are accompanied by a shift in the composition of symbiotic microbial communities [39,40,41]. Many researchers associate sea sponge diseases with changes in the composition of symbionts and the appearance of opportunistic infection resulting from changes in water temperature [42,43].

In this study, we have described the properties of the *Janthinobacterium* sp. SLB01 strain (*Oxalobacteraceae*) isolated from diseased *L. baicalensis* sponges. We have found that the SLB01 strain has proteolytic, lipolytic, and saccharolytic activities and can use citrate, lysine, ornithine, and arginine and reduce nitrates. The strain can use polysaccharides such as starch as a source of carbon; this was evidenced by the presence of amylase in the SLB01 genome [36]. The test for nitrate reductase was positive. Previously, it was shown that an increase in NO3+ can lead to a significant decrease in biofilm biomass in a nutrient-poor environment [44]. Moreover, the urease test in the SLB01 strain was negative, although its genome encodes a set of genes required for urea degradation [32]. Interestingly, the genomes of many *Janthinobacterium* spp. contain urease synthesis operons, but urease activity is not always detected. For example, in *Janthinobacterium lividum* ERGS5:01 and *Janthinobacterium violaceinigrum*, the test results were positive for urease [45,46], but they were negative for *Janthinobacterium tructae* and *Janthinobacterium psychrotolerans* [7,31].

We found that temperature affects violacein production and biofilm formation in the SLB01 strain. The concentration of violacein increases linearly on the fifth day at the temperature of 22 °C and remains constant, while the biofilm concentration continues to increase until the seventh day, followed by a gradual decrease. However, we revealed a sharp increase in biofilm formation at the temperature of 30 °C on the second day, with a subsequent fall. The reason for this phenomenon is not clear. The temperature of cultivation at 30 °C is critical for the survival of the strain, so neither bacterial biomass nor violacein increased during the cultivation. Bacteria probably begin to form a large amount of biofilm at this temperature to protect against aggressive environmental conditions [11].

At the same time, the production of violacein and biofilm constantly increases from the second to the tenth day at a temperature of 3 °C as the isolated strain is psychrophilic; the microorganisms and sponges live in the low temperatures of the Baikal water at 3–4 °C [35,38,47]. Moreover, violacein, as well as biofilms, represents a response to environmental stresses and is a key factor in the survival of these microorganisms [11]. It is known that biofilm development and violacein production are regulated by quorum-sensing systems [48,49]. We found that at 30 °C of cultivation, biofilm formation occurs without the production of violacein. The formation of biofilms in the absence of violacein production may indicate the complex nature of the induction.

In addition, the initial HPLC analysis allowed us to identify violacein and deoxyviolacein in the n-butanol extract of the *Janthinobacterium* SLB01 biomass, which constituted the bulk of the extract (53%), calculated from the area ratio of the peaks at 210 nm; the amount of deoxyviolacein was about 3.5% relative to the amount of violacein. MALDY TOF MS analysis confirmed the presence of violacein and deoxyviolacein in the crude extract. The extract contained peaks of pseudomolecular ions with *m*/*z* 344 [M + H]+ and 328 [M + H]+, which belong to violacein and deoxyviolacein, respectively.

Extraction of the concentrated butanol extract with chloroform allowed us to remove most of the unidentified compounds and substantially purify the desired violacein, but further experiments are needed to optimize the method, including optimization of the n-butanol/chloroform ratio and extraction temperature and extinction coefficient determination, as well as to accurately determine the compounds extractable with chloroform. Violacein can be easily isolated directly from the culture medium without the stages of destruction of bacterial cells and centrifugation, and it can be purified from the main impurities by extraction with chloroform. Among the substances that have a shorter retention time during chromatography, there may be metabolites of interest for biotechnology, for example, the tropodithietic acid antibiotic, the synthesis operon of which was identified in the analysis of the complete genome of the *Janthinobacterium* sp. SLB01 strain [36].

The results demonstrated that the SLB01 strain was resistant to multiple antibiotics. At the same time, there was sensitivity to various antibiotics such as gentamicin, kanamycin, tobramycin, doxycycline, tetracycline, and rifampicin. The analyzed strain is important in the realization of biological properties and adaptation to the environment.

Thus, in the present study, we have characterized in more detail the bacterial strain of *Janthinobacterium* sp. SLB01 from *L. baicalensis* sponge. Characteristics of the SLB01 strain make its properties an interesting addition to previous studies of the genome. We found a combination of twitching motility and biofilm formation that can be seen as factors facilitating Baikal sponge surface colonization. Moreover, violacein production can lead to a shift in the bacterial community of sponges and deterioration in their health. We expect that this study will contribute to the understanding of the relationships in microbial communities of sponges that are an important factor of the ecological situation of Lake Baikal.

## Figures and Tables

**Table 1:** Characteristics strain of the *Janthinobacterium* sp. SLB01 strain.

Characteristics	*Janthinobacterium* sp. Strain SLB01
Gram’s reaction	−
Motility	+
Nitrate reduction	+
H2S production	+
Urease production	−
Phenylalanine deamination	−
Lipolytic activity	+
Hydrolysis of gelatine	+
Starch	+
Casein	+
Arabinose	−
Glucose	+
Lactose	+
Mannose	+
Sucrose	+
Citrate	+
Adonitol	−
Inositol	+
D-Mannitol	−
D-Sorbitol	−
L-Arginine	+
L-Lysine	+
L-Ornithine	+

+ = Positive, − = negative.

**Table 2:** Antibiotic susceptibility test results of the *Janthinobacterium* sp. SLB01 strain by the disk diffusion method.

Antibiotic Tested	Symbol	Dose (µg)	Inhibition Zone Diameter (mm)	Interpretation
Ampicillin	AMP	10	5	R
Ceftriaxone	CTR	30	19	I
Imipenem	IPM	10	6	R
Nalidixic acid	NA	30	13	R
Levofloxacin	LE	5	15	R
Lomefloxacin	LOM	10	21	I
Norfloxacin	NX	10	5	R
Ofloxacin	OF	5	14	I
Ciprofloxacin	CIP	5	22	I
Amikacin	AK	30	22	S
Gentalyn	GEN	10	8	R
Gentamicin	HLG	120	16	S
Kanamycin	K	30	18	S
Netilmycin	NET	30	9	R
Tobramycin	TOB	10	15	S
Doxycycline	DO	30	30	S
Tetracycline	TE	30	25	S
Chloramphenicol	C	30	5	R
Erythromycin	E	15	5	R
Rifampicin	RIF	5	20	S

S—susceptible, I—intermediate, R—resistant.

## Data Availability

The data presented in this study are available on request from the corresponding author.
